# Comparison of Dosimetric Benefits of Three Precise Radiotherapy Techniques in Nasopharyngeal Carcinoma Patients Using a Priority-Classified Plan Optimization Model

**DOI:** 10.3389/fonc.2021.646584

**Published:** 2021-09-27

**Authors:** Qiaoli Wang, Jiyong Qin, Ruixue Cao, Tianrui Xu, Jiawen Yan, Sijin Zhu, Jiang Wu, Guoqiang Xu, Lixiu Zhu, Wei Jiang, Wenhui Li, Wei Xiong

**Affiliations:** ^1^ Department of Radiotherapy, Yunnan Cancer Hospital, the Third Affiliated Hospital of Kunming Medical University, Kunming, China; ^2^ Cancer Hospital Shenzhen Hospital, Chinese Academy of Medical Sciences and Peking Union Medical College, Shenzhen, China

**Keywords:** nasopharyngeal carcinoma, intensity-modulated radiotherapy, volumetric-modulated arc therapy, tomotherapy, dosimetry

## Abstract

**Introduction:**

Although intensity-modulated radiotherapy (IMRT), volumetric-modulated arc therapy (VMAT) and tomotherapy (TOMO) are broadly applied for nasopharyngeal carcinoma (NPC), the best technique remains unclear. Therefore, this study was conducted to address this issue.

**Methods:**

The priority-classified plan optimization model was applied to IMRT, VMAT and TOMO plans in forty NPC patients according to the latest international guidelines. And the dosimetric parameters of planning target volumes (PTVs) and organs at risk (OARs) were compared among these three techniques. The Friedman M test in SPSS software was applied to assess significant differences.

**Results:**

The median PGTVnx coverage of IMRT was the lowest (93.5%, P < 0.001) for all T categories. VMAT was comparable to TOMO in OARs clarified as priority I and II, and both satisfied the prescribed requirement. IMRT resulted in a relatively high dose for V25 and V30. Interestingly, subgroup analysis showed that the median PTV coverage of the three techniques was no less than 95% in the early T stage. The heterogeneity index (HI) of PGTVnx in VMAT was better than that in IMRT (P = 0.028). Compared to TOMO, VMAT showed a strong ability to protect eyesight and decrease low-dose radiation volumes. In the advanced T stage subgroup, TOMO numerically achieved the highest median PGTVnx coverage volume compared with VMAT and IMRT (93.61%, 91% and 90%, respectively). The best CI and HI of PCTV-1 were observed in TOMO. Furthermore, TOMO was better than VMAT for sparing the brain stem, spinal cord and temporal lobes (all P < 0.05). However, the median V5, V10, V15, V20 and V25 were significantly higher with TOMO than with VMAT (all P < 0.05).

**Conclusion:**

In the early T stage, VMAT provides a similar dose coverage and protection of OARs to IMRT, and there are no obvious advantages to choosing TOMO for NPC patients in the early T stage. TOMO may be recommended for patients in the advanced T stage due as it provides the largest dose coverage of PGTVnx and the best protection of the brain stem, spinal cord and temporal lobes. Additionally, more randomized clinical trials are needed for further clarification.

## Introduction

Nasopharyngeal carcinoma (NPC), one of the most common head and neck cancers, is commonly diagnosed in north Africa, southeast Asia and southern China (1, 2). According to global statistics published in 2018, approximately 129 thousand new cases occurred in 185 countries worldwide (3). NPC arises in a deep anatomical location, adjacent to many important organs, and tumor cells are extremely sensitive to radiotherapy. Accordingly, radiotherapy is an important means of anticancer therapy for NPC. Recent studies have shown that the 5-year survival rate of NPC patients ranges from 77.2% to 89.7%, with radiotherapy as the cornerstone of comprehensive treatment (4–6). However, because many important organs at risk (OARs) are adjacent to the nasopharynx, it is difficult to further improve local control of the tumor by increasing the radiation dose. Compared with the two-dimensional radiotherapy (2D-RT) technique, three-dimensional radiotherapy (3D-RT) technique has resulted in better survival rates, lower levels of damage to normal structures and better conformity of tumor targets in patients diagnosed with NPC (7, 8).

The three advanced radiation techniques commonly applied for NPC are intensity-modulated radiotherapy (IMRT), volumetric-modulated arc therapy (VMAT) and tomotherapy (TOMO). Although clinical practice has indicated that these three radiation techniques can meet the prescription dose requirement for NPC, the results of studies comparing the dosimetry of different radiation techniques in NPC are inconsistent. For example, He et al. (9) demonstrated that the VMAT plan was superior to the IMRT plan with regard to the dose distribution of targets and OAR protection. In contrast, another study concluded that the VMAT plan was the same as the IMRT plan in terms of sparing OARs (10), whereas other studies have shown that the TOMO plan shows dosimetric advantages over the IMRT plan (11, 12). Sun et al. (13) showed that VMAT was inferior to IMRT regarding the protection of OARs.

Nevertheless, few studies have focused on the comparison of IMRT, VMAT and TOMO plans for NPC. A study conducted by Lu et al. (14) showed that compared to the IMRT and VMAT plans, the TOMO plan achieved the best dosimetric parameters in the conformity index (CI), heterogeneity index (HI) and sparing of critical structures for NPC patients. In contrast, the maximum dose to the optic nerves, eyes and lens and the mean dose to the parotid glands and larynx were higher in TOMO than in VMAT. It is not clear which treatment would benefits NPC patients the most.

The biggest problem with most published studies is that they do not emphasize the priority of protecting OARs classified as priority I (brain stem, spinal cord, optic nerves and optic chiasma). In these studies, the coverage of planning targets was considered first when optimizing plans, which is contrary to the latest guidelines published in 2019 (15). Thus, the dose coverage of targets was more than 95%, but the dose of OARs classified as priority I (such as the spinal cord and brain stem) in some patients exceeded the guidelines. This is why the pass rate of the brain stem was as low as 28.8% (15/52) in Sun’s study (13) (the pass rate is defined as the percentage of patients meeting a prescribed dose limit). Hence, we designed this study to further illustrate the advantages and disadvantages of IMRT, VMAT and TOMO plans in patients diagnosed with NPC, based on the newest guidelines in which the priority of the dose coverage of the planning targets was lower than that of OARs classified as priority I. Additionally, because tumors in patients with advanced NPC are more adjacent to vital OARs, different radiotherapy techniques may have different dose distributions. Therefore, we used subgroup analysis to explore the best radiotherapy technique for patients in different clinical stages.

## Methods

### Patient Selection

Forty patients were randomly enrolled at the Radiation Department of Yunnan Cancer Hospital between January 2019 and March 2019 who were pathologically diagnosed with NPC and had received radiotherapy for the first time were included in this study. The treatment objective for all patients was to eradicate the tumor. According to the American Joint Committee on Cancer (AJCC) 8th edition system, the numbers of patients diagnosed with T1, T2, T3 and T4 stages were 8 (20.0%), 11 (27.5%), 12 (30%) and 9 (22.5%), respectively. There were 26 (65.0%) males and 14 (35.0%) females among the 40 included patients, with a median age of 50 years, ranging from 27 to 69 years.

All of the patients underwent magnetic resonance imaging (MRI) of the nasopharyngeal region and neck to guide the delineation of the target. Positron emission tomography-computed tomography (PET-CT) was applied when possible, considering the economic situation of the patients. In addition, detailed CT, nasopharyngoscopy and ultrasonography data for cervical lymph nodes were collected to interpret the location and extent of the tumor.

Our study was a retrospective analysis; patient details are not disclosed, and the patients were free to choose one of the three treatment plans (IMRT, VMAT and TOMO plans) for their treatment. Our study was reviewed and approved by the Ethics Committee of Yunnan Cancer Hospital. All the participants signed informed consent forms to participate in this study.

### Postural Immobilization and Treatment-Planning CT

The patients were immobilized with a head neck and shoulder thermoplastic mask in the supine position. Then, an enhanced computed tomography (CT) simulation scan was performed from the skull vertex to 2 centimeters below the sternoclavicular joint. The CT images transferred to the treatment planning system (TPS) were reconstructed with a 3-millimeter slice thickness. Each treatment plan was replanned for IMRT, VMAT and TOMO based on the same set of CT images.

### Delineation of Target Volumes and Organs at Risk

For all patients, target volumes were delineated by a senior radiation oncologist to avoid differences resulting from the approach of different clinicians. Standard delineation of the target volume referred to International Commission on Radiation Units and Measurements reports (ICRU) 50, 62 and 83. When the three recommendations were inconsistent, we adopted the latest one. MRI, CT, nasopharyngoscopy and PET-CT were employed to guide delineation of the gross tumor volume (GTV). GTVnx was defined as the primary tumor location and posterior pharyngeal lymph nodes. GTVnd was defined as neck lymph nodes distinguished by imaging while regardless of whether they were positive or negative.

The clinical target volumes (CTVs) included CTV-1 and CTV-2. Notably, the delineation of CTVs in different radiotherapy centers varies, and in our study, we followed the recommendation of Lee et al. (16). CTV-1, defined as high-risk areas, was formed by three-dimensional space expansion of 5 mm based on GTVnx. CTV-2, defined as medium- and low-risk areas, was formed by three-dimensional space expansion of 5 mm based on CTV-1. Additionally, both CTV-1 and CTV-2 were manually modified to cover the area of potential invasion which may including the vascular sheath, natural channel of the skull base and other vulnerable substructures.

The positioning error at our center is set as 3 mm; thus, various planning target volumes (PTVs) were defined by uniformly expanding 3 mm in 6 axes on the basis of the respective target volumes and then manually modifying the PTVs to avoid covering vital OARs, corresponding to PGTVnx, PGTVnd, PCTV-1 and PCTV-2. To avoid acute dermal toxicity, all PTVs were reduced to 3 mm below the skin surface.

The relevant OARs were classified into 4 priority levels according to the latest guidelines (15), as follows: (a) priority I OARs, defined as critical normal structures, including the brain stem, spinal cord, optic chiasma and optic nerve; (b) priority II OARs including the temporal lobes; (c) priority III OARs including the eye, lens and pituitary gland; and (d) priority IV OARs including the parotid gland, mandible, temporal-mandibular joint (TMJ), thyroid, inner ear and oral cavity.

To better protect critical OARs, all priority I OARs, including the temporal lobes but excluding the spinal cord, were extended to 3-mm margins *via* 3D expansion to form the planning risk volume (PRV)-brain stem, PRV-optic chiasma and PRV-optic nerves. The PRV-spinal cord was defined from the spinal cord extending to 5-mm margins with 3D expansion.

### Prescription Dose

The prescription dose for PTVs was designed as three levels in 33 fractions with simultaneous integrated boosts (SIBs). PGTVnx, PGTVnd, PCTV-1 and PCTV-2 received 69.96 Gy, 69.96 Gy, 59.4 Gy and 54 Gy, respectively.

### Dose Restriction on Organs at Risk

The dosimetric restriction of OARs was based on the latest international guidelines for NPC in which the OARs are divided into four priority levels (15); details are shown in **Table 1**. The desirable approximate maximum dose (D_0.03cc_) of the PRV-brain stem, PRV-optic chiasma and PRV-optic nerves was no more than 54 Gy. If the tumor is particularly close to these OARs, which may lead to a serious dose loss in the PTV, the maximum acceptance criteria (MAC) of the actual volume of the OARs can be relaxed to no more than 60 Gy. In addition, the desirable maximum dose of PRV-spinal cord was no more than 45 Gy, while the MAC of the spinal cord was no more than 50 Gy.

**Table 1:** Dose restriction on organs at risk.

OARs	Priority level	Desirable dose	MAC
Brain stem	1	D_0.03cc_PRV _≤_ 54 Gy	≤60 Gy
Optic chiasma	1	D_0.03cc_PRV _≤_ 54 Gy	≤60 Gy
Optic nerves	1	D_0.03cc_PRV _≤_ 54 Gy	≤60 Gy
Spinal cord	1	D_0.03cc_PRV _≤_ 45 Gy	≤50 Gy
Temporal lobes	2	T_1-2_: D_0.03cc_PRV _≤_ 65 Gy	/
		T_3-4_: D_0.03cc_PRV _≤_ 70 Gy	≤72 Gy
Lenses	3	D_0.03cc ≤_ 6 Gy	D_0.03cc ≤_ 15 Gy
Eyes	3	D_mean_ ≤ 35 Gy	D_0.03cc ≤_ 50 Gy
Pituitary gland	3	D_0.03cc ≤_ 60 Gy	D_0.03cc ≤_ 65 Gy
Parotid glands	4	D_mean_ ≤ 26 Gy	V30<50% (at least one side)
Mandible	4	D2 ≤ 70 Gy	≤75 Gy
TMJs	4	D2 ≤ 70 Gy	≤75 Gy
Inner ears	4	D_mean_ ≤ 45 Gy	≤55 Gy
Oral cavity	4	D_mean_ ≤ 40 Gy	≤50 Gy
Thyroid gland	4	V50 ≤ 60%	V60 ≤ 10cm^3^

OARs, organs at risk; MAC, maximum acceptance criteria; TMJs, temporal-mandibular joints, PRV, planning target volume; D_0.03cc_, an approximate maximum dose for the organs at risk; D_mean_, the mean dose of the organs at risk; D50, dose received by 50% of the volume; D2, dose received by 2% of the volume; V50, the volume of which received 50 Gy; V60, the volume of which received 60 Gy.

Of note, desirable approximate maximum dose limits of the PRV-temporal lobes were associated with tumor T staging; thus, the dose limitation of T_1-2_ was no more than 65 Gy and that of T_3-4_ was no more than 70 Gy. For the T_3-4_ stage, the MAC of the temporal lobes was no more than 72 Gy.

### Principles of Priority-Classified Plan Optimization

All plans adhered to the international guidelines on dose prioritization and acceptance criteria published in 2019, which states that PTV coverage should consider critical OARs to avoid highly morbid sequelae or potentially lethal damage. Hence, all plans in our study first conformed to the limitations of OARs classified as priority I (brain stem, optic nerves, optic chiasma and spinal cord). Then the items classified as priority II were considered, including temporal lobes. The dose coverage of the PTVs were also classified as priority II, which means PTVs must give ways to OARs classified as priority I. Finally, dose limitations for OARs classified as priority III and IV were considered in order of priority as much as possible.

### Principles of PTVs’ Dose Coverage

The dose coverage requirements of PGTVnx and PGTVnd were normalized as follows: (a) the volume of 95% PTVs received 100% of the prescription dose, (b) no more than 20% of PTVs received more than 110% of the prescription dose, (c) no more than 5% of PTVs received more than 115% of the prescription dose and (d) no more than 1% of PTVs received less than 93% of the prescription dose. Moreover, PCTV-1 and PCTV-2 require both (a) and (d).

### Planning Objectives and Techniques

The IMRT, VMAT and TOMO plans for each included patient were completed by the same medical physicist. One senior medical physicist was responsible for all of the plans, which were delivered using a 6-MV X-ray beam. Additionally, the PTV and OAR doses were optimized to the same level based on the principles referred to above.

The IMRT and VMAT plans were generated using the pinnacle (version 9.1, Philips, Inc., USA) treatment planning system (TPS). The IMRT plan was generated using step and shoot techniques with coplanar 9 field IMRT (Elekta-VersaHD) based on 160 multileaf collimators (MLCs). The dose grid, maximum segment number, minimum segment area and monitor units (MUs) were set to 0.3 cm, 120, 4 cm^2^ and 4 MU, respectively. The VMAT plan was generated using two arcs (one clockwise from +180° to -180° and one counterclockwise from -180° to +180°) with a total of 182 control points based on Elekta-VersaHD. For the IMRT plan, the optimization algorithm was direct machine parameter optimization (DMPO), the calculation algorithm for the intermediate dose during the optimization process was TPB, and the calculation algorithm for the final dose was collapsed cone convolution superposition (CCCS); for the VMAT plan, the optimization algorithm was SmartArc, the calculation algorithm for the intermediate dose during the optimization process was singular value decomposition (SVD), and the final dose calculation algorithm was CCCS. Furthermore, the optimization process was as consistent as possible. The iterations of all plans were optimized for 160 with artificial intervention point after every 40 iterations.

In addition, TOMO used X-rays in 6MV FFF mode and the tomotherapy planning station (Hi-Art Version 3.2.3.2, Madison, WI) was used for the TOMO plan, and the three major parameters were as follows: field width, 2.512 cm; pitch, 0.2; and modulation factor, 3.5.

### Planning Comparison

The dosimetric parameters applied to evaluate the PTVs included V69.96 (the volume of the PGTVnx covered by the 69.96 Gy isodose), V59.4 (the volume of the PCTV-1 covered by the 59.4 Gy isodose), V54 (the volume of the PCTV-2 covered by the 54 Gy isodose), D2 (D2 is defined as the approximate maximum dose), D50, D95 and D98 (D98 is defined as the approximate minimum dose), CI and the heterogeneity index (HI). D50 and D95 were defined as the doses covering 50% and 95% of the PTVs, respectively. CI was calculated as CI=TV_PIV_/TV × TV_PIV_/PIV (TV_PIV_: the target volume covered by the prescription isodose; TV: the target volume; PIV: the volume of the reference isodose) according to research conducted by Paddick (17). The CI values closer to 1 indicated that the plan is more conformable. HI was calculated as HI=(D2-D98)/D50, in accordance with the method published by Sun et al. (13). The closer HI is to 0, the better the homogeneity is.

The MAC dose was one of the indicators for which all OARs needed to be evaluated. In addition, different OARs were evaluated with different dosimetric parameters. D_0.03cc_PRV was used to analyze the brain stem, spinal cord, optic chiasma, temporal lobe and optic nerve. The mean doses to the eye, parotid glands, inner ear and oral cavity were analyzed. The doses covering a 2% volume (D2) of the mandible and TMJ, the relative volume of the parotid gland receiving more than 30 Gy and the relative volume of the thyroid gland receiving more than 60 Gy were also examined.

We also focused on the low-dose radiation volume of the body, as calculated as the volume of the body receiving more than 5 Gy, 10 Gy, 15 Gy, 20 Gy, 25 Gy and 30 Gy (V5, V10, V15, V20, V25 and V30).

### Statistical Analysis

SPSS 24.0 software (SPSS Inc., Chicago, IL) was employed to perform the statistical analysis. Differences among IMRT, VMAT and TOMO were compared through the Friedman M test. If there were significant differences among the three plans, the Friedman M test was used again to compare any two of the three plans. A two-tailed P value less than 0.05 was considered a significant difference.

## Results

### Dosimetric Parameters of PTVs

As indicated in **Table 2**, the overall results showed that the median V69.96 (the volume of the PGTVnx covered by the 69.96 Gy isodose) of the VMAT plan and TOMO plan was similar, with neither lower than 95% (95% *vs.* 95.24%, P = 0.656). The median value for the V69.96 in the IMRT plan was significantly the lowest, and reached as low as 93.5%.

**Table 2:** Dosimetric comparison of IMRT, VMAT and TOMO for PTVs in 40 NPC patients.

		Median (IQR)				P-value		
Target	Index	IMRT	VMAT	TOMO	P	I *vs.* V	I *vs.* T	V *vs.* T
PGTVnx	V69.96 (%)	93.50 (90.00-95.00)	95.00 (90.25-97.00)	95.24 (93.59-97.54)	<0.001	0.004	<0.001	0.656
	D2 (Gy)	75.77 (74.45-76.36)	74.37 (73.60-77.36)	75.38 (74.66-76.23)	0.273	_	_	_
	D50 (Gy)	72.64 (72.03-73.02)	72.21 (71.83-73.15)	73.30 (72.53-73.73)	0.023	0.438	0.596	0.018
	D95 (Gy)	69.56 (68.21-70.03)	69.90 (67.25-70.34)	70.02 (69.46-70.48)	<0.001	0.016	<0.001	0.221
	D98 (Gy)	67.81 (63.86-69.11)	67.73 (62.19-69.63)	68.80 (66.32-69.74)	<0.001	0.281	<0.001	0.057
	CI	0.435 (0.323-0.498)	0.445 (0.355-0.500)	0.474 (0.332-0.525)	0.026	0.221	0.03	1
	HI	0.110 (0.080-0.160)	0.125 (0.060-0.205)	0.087 (0.078-0.140)	0.074	_	_	_
PCTV-1	V59.4 (%)	99.00 (98.00-99.00)	98.50 (97.00-99.00)	98.96 (98.27-99.62)	0.581	_	_	_
	D2 (Gy)	75.47 (74.24-76.09)	74.20 (73.38-76.92)	75.16 (74.57-76.07)	0.103	_	_	_
	D50 (Gy)	71.38 (70.54-72.10)	71.26 (70.70-72.08)	71.75 (70.57-72.92)	0.139	_	_	_
	D95 (Gy)	62.36 (61.55-63.70)	62.79 (61.51-63.81)	62.75 (61.06-63.74)	0.622	_	_	_
	D98 (Gy)	60.03 (59.11-61.30)	60.66 (58.03-61.89)	60.77 (59.63-61.65)	0.22	_	_	_
	CI	0.325 (0.250-0.378)	0.350 (0.253-0.398)	0.385 (0.333-0.460)	<0.001	0.008	<0.001	0.036
PCTV-2	V54 (%)	98.00 (96.00-98.00)	97.50 (97.00-99.00)	98.09 (96.46-98.87)	0.135	_	_	_
	D2 (Gy)	74.63 (73.47-75.37)	73.56 (73.00-75.86)	74.52 (74.21-75.52)	0.098	_	_	_
	D50 (Gy)	64.12 (61.90-65.64)	64.37 (61.88-66.09)	62.27 (60.16-64.39)	<0.001	1	<0.001	<0.001
	D95 (Gy)	55.13 (54.96-55.47)	55.05 (54.64-55.42)	55.31 (54.70-55.87)	0.265	_	_	_
	D98 (Gy)	55.53 (52.62-54.10)	53.66 (52.43-54.33)	54.14 (52.56-54.90)	0.153	_	_	
	CI	0.690 (0.670-0.748)	0.760 (0.733-0.803)	0.730 (0.690-0.770)	<0.001	<0.001	0.005	0.009

IMRT/I, intensity modulated radiation therapy; VMAT/V, volumetric modulated arc therapy; TOMO/T, tomotherapy; Gy, gray; IQR, inter-quartile range; DV, the absorbed dose in v% of the volume; CI, conformity index; HI, homogeneity index; Vx, the volume of organ receiving more or equal to x Gy.

Additionally, the D50, D95 and D98 were significantly different among the three plans for PGTVnx (P = 0.023, P < 0.001, P < 0.001, respectively). The D2, D95, D98 and HI of the three PTVs were similar among IMRT, VMAT and TOMO. Interestingly, the CIs of the PGTVnx, PCTV-1 and PCTV-2 showed significant differences. The CIs of PGTVnx, PCTV-1 and PCTV-2 in the IMRT were the worst among the three plans.

### Dosimetric Parameters of OARs

**Table 3** and **Supplementary Table 1** show the results of OARs sparing.

**Table 3:** Dosimetric comparison of IMRT, VMAT and TOMO for organs at risk (priority level I and II) in 40 NPC patients.

		Median (IQR)				P-value		
OAR	Objective	IMRT	VMAT	TOMO	P	I *vs.* V	I *vs.* T	V *vs.* T
Brain stem	D_max_ (Gy)	53.13 (49.23-55.29)	53.74 (49.24-57.16)	51.74 (44.74-54.34)	<0.001	0.791	0.016	<0.001
Brain stem_PRV	D_0.03cc_ (Gy)	56.50 (52.31-60.34)	58.18 (52.78-62.96)	58.67 (54.19-63.31)	0.407	_	_	_
Optic chiasm	D_max_ (Gy)	27.51 (10.24-45.85)	27.88 (10.03-44.05)	31.86 (20.90-45.82)	0.004	1	0.03	0.005
Optic chiasma_PRV	D_0.03cc_ (Gy)	36.77 (18.73-53.54)	41.40 (18.39-51.75)	41.67 (28.14-54.36)	<0.001	0.353	0.03	<0.001
Optic nerve_L	D_max_ (Gy)	16.25 (7.87-38.55)	19.41 (7.72-37.70)	29.17 (18.35-42.68)	<0.001	1	<0.001	<0.001
Optic nerve_L PRV	D_0.03cc_ (Gy)	26.87 (12.27-46.83)	30.73 (13.24-50.25)	35.87 (25.45-52.81)	<0.001	0.943	0.001	<0.001
Optic nerve_R	D_max_ (Gy)	17.08 (8.07-40.61)	19.46 (7.83-39.03)	32.86 (18.12-44.37)	<0.001	1	0.002	0.001
Optic nerve_R PRV	D_0.03cc_ (Gy)	31.22 (13.11-48.98)	30.32 (13.07-50.97)	41.74 (26.82-53.22)	<0.001	0.353	0.03	<0.001
Spinal cord	D_max_ (Gy)	39.12 (37.99-40.36)	40.29 (38.20-41.98)	35.57 (32.48-38.20)	<0.001	0.101	<0.001	<0.001
Spinal cord_PRV	D_0.03cc_ (Gy)	44.12 (42.13-47.48)	44.46 (43.00-48.08)	43.31 (40.16-49.35)	0.163	_	_	_
Temporal lobe_L	D_max_ (Gy)	73.73 (64.63-77.21)	73.16 (66.83-78.16)	72.10 (64.34-75.94)	<0.001	1	<0.001	<0.001
Temporal lobe_L PRV	D_0.03cc_ (Gy)	75.25 (71.47-77.00)	74.77 (71.69-78.34)	73.63 (69.29-75.95)	<0.001	1	<0.001	<0.001
Temporal lobe_R	D_max_ (Gy)	75.74 (67.58-77.54)	74.30 (68.85-77.92)	73.27 (64.84-75.84)	<0.001	1	<0.001	<0.001
Temporal lobe_R PRV	D_0.03cc_ (Gy)	76.34 (72.47-77.60)	75.06 (72.20-79.19)	74.34 (71.68-75.64)	0.001	1	0.002	0.009

IMRT/I, intensity modulated radiation therapy; VMAT/V, volumetric modulated arc therapy; TOMO/T, tomotherapy; OAR, organ at risk; IQR, inter-quartile range; Gy, gray; PRV, planning risk volume; L, left; R, right; Dmax, the maximum dose; D0.03cc, an approximate maximum dose for the organs at risk.

For most of the OARs, such as brain stem, optic chiasma, spinal cord, temporal lobe, lens, TMJ, oral cavity and thyroid gland, there were no significant differences between IMRT and VMAT. TOMO was superior for sparing of the temporal lobe, spinal cord, brain stem and oral cavity. However, TOMO resulted in significantly the highest dose delivered to optic chiasma, optic nerve and pituitary gland. Regarding the D_0.03cc_ of the pituitary gland, TOMO delivered the highest dose and VMAT delivered the lowest dose. Concerning the ability to protected the parotid gland, the TOMO plan was comparable to the VMAT plan.

### Comparison of Low Dose Radiation Volume in the Body

**Table 4** shows the low dose radiation volumes of the body for the three plans.

**Table 4:** Low-dose radiation volume of IMRT, VMAT and TOMO in 40 NPC patients.

		Median (IQR)				P-value		
OAR	Objective	IMRT (cm^3^)	VMAT (cm^3^)	TOMO (cm^3^)	P	I *vs.* V	I *vs.* T	V *vs.* T
Body	V5	6231 (5313–7253)	6371 (5398-7248)	6348 (5113-7183)	0.592	_	_	_
	V10	5110 (4454-6166)	5181 (4426-6067)	5490 (4600-6302)	<0.001	0.791	<0.001	<0.001
	V15	4522 (3865-5436)	4499 (3825-5419)	4691 (3945-5649)	<0.001	0.281	<0.001	<0.001
	V20	3949 (3391-4772)	3957 (3316-4847)	4114 (3370-5086)	<0.001	0.656	<0.001	<0.001
	V25	3519 (2952-4369)	3485 (2889-4300)	3471 (2796-4514)	0.001	0.004	1	0.002
	V30	3075 (2523-3970)	2980 (2468-3807)	2909 (2279-3921)	<0.001	<0.001	0.011	0.353

IMRT/I, intensity modulated radiation therapy; VMAT/V, volumetric modulated arc therapy; TOMO/T, tomotherapy; OAR, organ at risk; IQR, inter-quartile range; Vx, the volume of organ receiving more or equal to x Gy.

The low-dose volume of healthy tissue was significantly the highest in the TOMO plan regarding V10, V15 and V20 (all P < 0.01, Vx defined as the volume of body that received more than xGy), while these parameters were comparable in the IMRT plan and the VMAT plan. In addition, the values of V25 and V30 with the IMRT plan were significantly higher than those with the VMAT plan (all P < 0.05). In conclusion, the TOMO plan had no obvious advantages among the three plans.

### Subgroup Analysis: Comparison of the Three Plans for T_1-2_-Stage Patients

**Figure 1:**
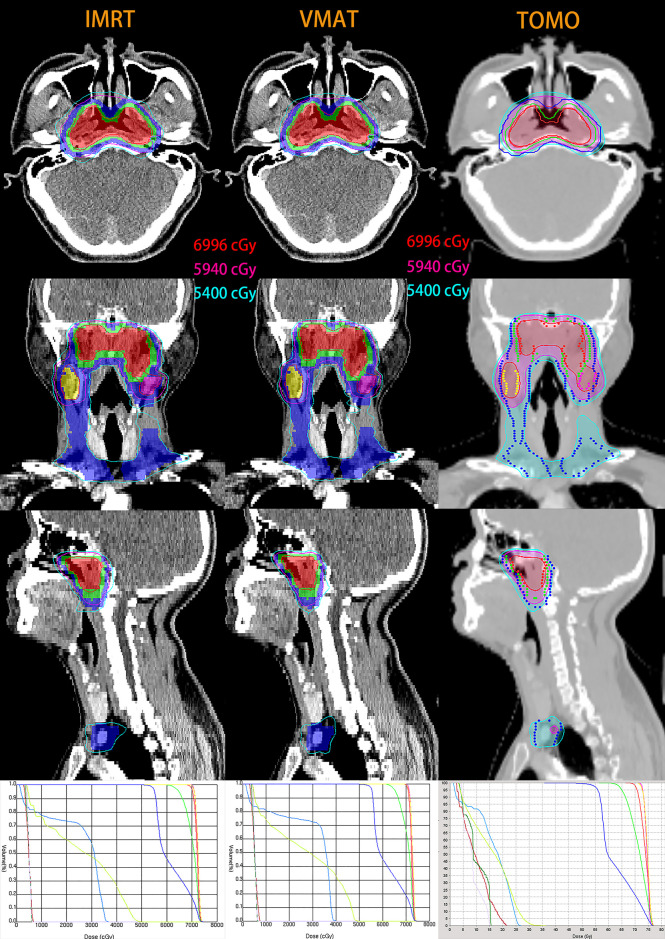
Isodose distributions and dose-volume histograms (DVHs) for a representative T_1_-stage NPC patient with IMRT (left), VMAT (middle) and TOMO (right) planning. Maroon, forest, lavender, yellow-green, light-blue, red, green and blue lines in three DVH are optic nerve in left, optic nerve in right, optic chiasma, brain stem, spinal cord, PGTVnx, PCTV-1 and PCTV-2, respectively. For IMRT and VMAT planning, color-wash areas: PGTVnx (red), PGTVnd-left (purple), PGTVnd-right (yellow), PCTV-1 (green), PCTV-2 (blue); and the red, purple and sky-blue lines are isodose curves of 69.96Gy, 59.4Gy and 54Gy. For TOMO planning, isodose curves of 69.96Gy, 59.4Gy and 54Gy are shaded in the red, purple and sky-blue, respectively; targets are represented by lines: PGTVnx (red), PGTVnd-left (purple), PGTVnd-right (yellow), CTV-1 (green), PCTV-2 (blue).

**Figure 1** depicts the isodose distributions and dose-volume histograms (DVHs) for a representative T_1_-stage NPC patient planned by IMRT, VMAT and TOMO. **Table 5** shows the dosimetric parameters of PTVs and **Table 6** and **Supplementary Table 2** show the results of OARs sparing in T_1-2_-stage patients.

**Table 5:** Comparison of targets in T_1-2_ NPC patients.

		Median (IQR)				P-value		
Target	Index	IMRT	VMAT	TOMO	P	I *vs.* V	I *vs.* T	V *vs.* T
PGTVnx	V69.96 (%)	95.00 (95.00-96.00)	97.00 (96.00-98.00)	97.42 (96.22-98.05)	<0.001	0.004	<0.001	1
	D2 (Gy)	74.54 (74.00-75.45)	73.71 (73.16-74.43)	75.13 (74.55-75.75)	0.076	_	_	_
	D50 (Gy)	72.24 (71.99-72.95)	71.85 (71.61-72.29)	73.28 (72.28-73.56)	0.002	0.045	0.991	0.002
	D95 (Gy)	69.97 (69.81-70.15)	70.35 (70.03-70.47)	70.47 (70.16-70.66)	<0.001	0.006	0.001	1
	D98 (Gy)	69.11 (68.27-69.34)	69.68 (69.36-69.89)	69.79 (69.29-69.97)	<0.001	0.011	<0.001	0.991
	CI	0.330 (0.270-0.420)	0.370 (0.240-0.430)	0.325 (0.274-0.473)	0.128	_	_	_
	HI	0.080 (0.070-0.100)	0.060 (0.050-0.080)	0.078 (0.062-0.086)	0.032	0.028	0.875	0.37
PCTV-1	V59.4 (%)	99.00 (98.00-99.00)	99.00 (98.00-100.00)	99.21 (97.93-99.80)	0.22	_	_	_
	D2 (Gy)	74.18 (73.45-75.10)	73.51 (72.89-74.03)	74.88 (74.42-75.41)	0.003	0.017	1	0.006
	D50 (Gy)	70.52 (69.97-70.96)	70.69 (70.24-71.09)	70.53 (69.90-71.00)	0.532	_	_	_
	D95 (Gy)	62.62 (61.49-64.29)	63.41 (62.03-64.07)	61.71 (60.55-62.27)	0.014	0.433	0.433	0.011
	D98 (Gy)	60.63 (59.90-61.96)	61.52 (60.68-62.29)	60.29 (59.30-61.41)	0.076	_	_	_
	CI	0.250 (0.230-0.300)	0.270 (0.240-0.350)	0.330 (0.210-0.360)	0.002	0.036	0.003	1
PCTV-2	V54 (%)	98.00 (97.00-98.00)	98.00 (97.00-99.00)	97.83 (96.53-98.82)	0.336	_	_	_
	D2 (Gy)	73.46 (72.83-74.41)	73.12 (72.63-73.54)	74.32 (73.77-75.15)	0.05	_	_	_
	D50 (Gy)	62.57 (61.39-64.19)	62.49 (60.61-64.26)	60.90 (58.39-63.15)	0.004	0.768	0.004	0.105
	D95 (Gy)	55.19 (55.01-55.49)	55.10 (54.63-55.40)	55.10 (54.68-55.86)	0.692	_	_	_
	D98 (Gy)	53.75 (53.45-54.25)	54.05 (53.24-54.54)	54.08 (52.86-54.60)	0.504	_	_	_
	CI	0.700 (0.680-0.750)	0.760 (0.750-0.810)	0.730 (0.670-0.780)	<0.001	<0.001	0.875	0.004

IMRT/I, intensity modulated radiation therapy; VMAT/V, volumetric modulated arc therapy; TOMO/T, tomotherapy; Gy, gray; IQR, inter-quartile range; DV, the absorbed dose in v% of the volume; CI, conformity index; HI, homogeneity index; Vx, the volume of organ receiving more or equal to x Gy.

**Table 6:** Comparison of organs at risk (priority level I and II) in T_1-2_ NPC patients.

		Median (IQR)				P-value		
OAR	Objective	IMRT	VMAT	TOMO	P	I *vs.* V	I *vs.* T	V *vs.* T
Brain stem	D_max_ (Gy)	49.63 (47.56-54.50)	50.92 (48.46-55.73)	48.60 (41.52-53.40)	0.04	0.871	0.035	0.023
Brain stem_PRV	D_0.03cc_ (Gy)	52.87 (51.49-57.46)	53.43 (51.97-58.76)	56.67 (51.70-59.55)	0.809	_	_	_
Optic chiasm	D_max_ (Gy)	10.00 (8.52-15.32)	9.58 (8.14-12.47)	20.88 (15.30-30.29)	<0.001	0.433	0.011	<0.001
Optic chiasma_PRV	D_0.03cc_ (Gy)	18.56 (12.59-25.25)	18.31 (12.03-23.44)	28.75 (23.77-40.12)	<0.001	0.433	0.002	<0.001
Optic nerves_L	D_max_ (Gy)	7.76 (5.89-10.51)	7.69 (5.81-9.38)	18.02 (13.90-21.37)	<0.001	1	<0.001	<0.001
Optic nerves_L PRV	D_0.03cc_ (Gy)	11.80 (9.22-17.65)	13.08 (8.86-16.17)	24.80 (20.87-29.45)	0.001	1	0.017	0.001
Optic nerves_R	D_max_ (Gy)	7.84 (6.57-10.78)	7.61 (6.78-11.29)	17.05 (13.04-24.58)	<0.001	1	0.002	0.002
Optic nerves_R PRV	D_0.03cc_ (Gy)	13.10 (9.76-20.09)	12.11 (10.63-21.35)	26.59 (20.93-31.58)	<0.001	0.991	0.011	<0.001
Spinal cord	D_max_ (Gy)	38.75 (37.77-40.18)	39.66 (38.10-41.28)	34.01 (32.42-37.72)	<0.001	1	0.001	<0.001
Spinal cord_PRV	D_0.03cc_ (Gy)	43.20 (42.35-44.90)	43.16 (42.49-44.74)	42.06 (39.82-44.09)	0.018	1	0.069	0.028
Temporal lobe_L	D_max_ (Gy)	64.87 (62.92-71.83)	66.44 (61.54-70.85)	64.21 (58.37-67.55)	0.001	1	0.004	0.006
Temporal lobe_L PRV	D_0.03cc_ (Gy)	71.21 (69.42-73.77)	71.49 (67.84-73.06)	68.95 (64.28-72.10)	0.001	1	0.001	0.017
Temporal lobe_R	D_max_ (Gy)	67.31 (62. 72-72.40)	68.56 (61.94-71.97)	64.80 (58.73-70.46)	0.018	1	0.028	0.069
Temporal lobe_R PRV	D_0.03cc_ (Gy)	72.31 (68.95-74.14)	72.01 (67.95-73.21)	71.60 (67.18-73.09)	0.065	_	_	_

IMRT/I, intensity modulated radiation therapy; VMAT/V, volumetric modulated arc therapy; TOMO/T, tomotherapy; OAR, organ at risk; IQR, inter-quartile range; Gy, gray; PRV, planning risk volume; L, left; R, right; Dmax, the maximum dose; D0.03cc, an approximate maximum dose for the organs at risk.

Subgroup analysis in cases at T_1-2_-stage demonstrated that the IMRT, VMAT and TOMO plans resulted in no less than 95% volume of the prescription dose coverage for all PTVs. The median V69.96 was significantly lower for the IMRT plan than for the VMAT or TOMO plan (P = 0.004 and P < 0.001, respectively) (**Table 5**). However, the median V59.4 and V54 were similar among the IMRT, VMAT and TOMO plans (P > 0.05).

In patients with the T_1-2_-stage cancer, there were no significant differences among the three radiation techniques in D2 and CI of the PGTVnx (all P > 0.05). TOMO achieved the highest dose in the D50, D95 and D98 of the PGTVnx. Furthermore, the HI of the PGTVnx in the VMAT plan was significantly superior to that in the IMRT plan. TOMO resulted in the best CI of PCTV-1. In the PCTV-2, the three plans showed no significant difference in D2, D95 and D98.

The dose delivered to the optic chiasma, optic nerve, pituitary gland and the D_0.03cc_ of the eye in the TOMO plan were the highest. In contrast, the TOMO plan significantly showed the best ability to protect the brain stem, spinal cord and temporal lobes. Regarding the lens, parotid gland, mandible, TMJ, inner ear, oral cavity and thyroid gland, no significant difference was observed among the three plans.

As was shown in the **Table 7**, the low-dose volume of healthy tissue was significantly the highest in the TOMO plan regarding V5, V10, V15 and V20 (all P < 0.05). The V5, V10 and V15 of IMRT were comparable to those of the VMAT plan. In addition V30 was the highest in the IMRT plan.

**Table 7:** Low-dose radiation volume of IMRT, VMAT and TOMO in T_1-2_ NPC patients.

		Median (IQR)				P-value		
OAR	Objective	IMRT (cm^3^)	VMAT (cm^3^)	TOMO (cm^3^)	P	I *vs.* V	I *vs.* T	V *vs.* T
Body	V5	5726 (4576-6701)	5659 (4548-6905)	5769 (4706-7105)	<0.001	0.433	<0.001	0.045
	V10	4642 (3794-5479)	4531 (3782-5501)	4896 (3796-5825)	0.001	1	0.006	0.004
	V15	4014 (3363-4654)	3944 (3277-4577)	4207 (3325-4900)	<0.001	0.314	0.028	<0.001
	V20	3404 (2946-3960)	3469 (2900-3978)	3638 (2894-4173)	0.002	0.991	0.045	0.002
	V25	2973 (2572-3510)	2987 (2509-3476)	2932 (2445-3440)	0.065	_	_	_
	V30	2566 (22219-3017)	2524 (2073-2933)	2321 (1950-2806)	0.001	0.006	0.004	1

IMRT/I, intensity modulated radiation therapy; VMAT/V, volumetric modulated arc therapy; TOMO/T, tomotherapy; OAR, organ at risk; IQR, inter-quartile range; Vx, the volume of organ receiving more or equal to x Gy.

### Subgroup Analysis: Comparison of the Three Plans for T_3-4_-Stage Patients

**Figure 2:**
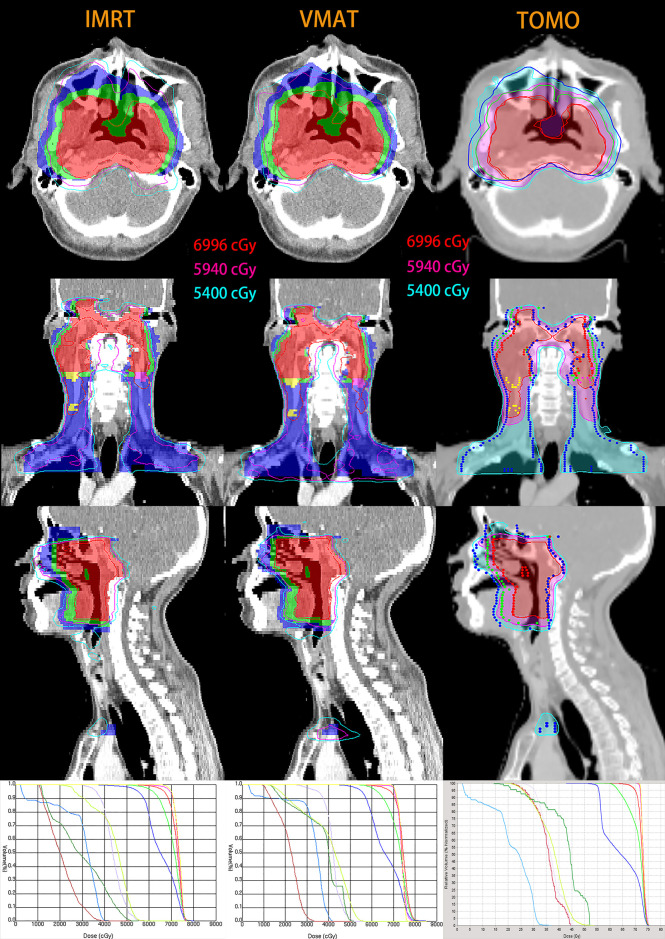
Isodose distributions and dose-volume histograms (DVHs) for a representative T_4_-stage NPC patient with IMRT (left), VMAT (middle) and TOMO (right) planning. Maroon, forest, lavender, yellow-green, light-blue, red, green and blue lines in three DVH are optic nerve in left, optic nerve in right, optic chiasma, brain stem, spinal cord, PGTVnx, PCTV-1 and PCTV-2, respectively. For IMRT and VMAT planning, color-wash areas: PGTVnx (red), PGTVnd-left (purple), PGTVnd-right (yellow), PCTV-1 (green), PCTV-2 (blue); and the red, purple and sky-blue lines are isodose curves of 69.96Gy, 59.4Gy and 54Gy. For TOMO planning, isodose curves of 69.96Gy, 59.4Gy and 54Gy are shaded in the red, purple and sky-blue, respectively; targets are represented by lines: PGTVnx (red), PGTVnd-left (purple), PGTVnd-right (yellow), PCTV-1 (green), PCTV-2 (blue).

The isodose distributions and dose-volume histograms (DVHs) for a representative T_4_-stage NPC patient planned by IMRT, VMAT and TOMO are illustrated in **Figure 2**. **Table 8** shows the dosimetric parameters of PTVs and **Table 9** and **Supplementary Table 3** show the results of OARs sparing.

**Table 8:** Comparison of targets in T_3-4_ NPC patients.

		Median (IQR)				P-value		
Target	Index	IMRT	VMAT	TOMO	P	I *vs.* V	I *vs.* T	V *vs.* T
PGTVnx	V69.96 (%)	90.00 (87.50-93.00)	91.00 (90.00-95.00)	93.61 (91.90-94.83)	0.037	0.495	0.033	0.741
	D2 (Gy)	76.08 (75.82-76.78)	76.66 (74.29-78.58)	75.75 (74.79-76.93)	0.651	_	_	_
	D50 (Gy)	72.81 (72.20-73.26)	72.90 (72.04-74.02)	73.35 (72.51-73.95)	0.688	_	_	_
	D95 (Gy)	68.24 (66.43-69.07)	67.79 (64.98-69.71)	69.49 (68.35-69.90)	0.018	1	0.016	0.192
	D98 (Gy)	63.92 (60.43-67.14)	63.53 (58.69-66.58)	66.89 (61.43-68.12)	0.06	_	_	_
	CI	0.490 (0.440-0.540)	0.500 (0.445-0.540)	0.507 (0.468-0.574)	0.165	_	_	_
	HI	0.160 (0.115-0.225)	0.190 (0.130-0.245)	0.135 (0.101-0.197)	0.091	_	_	_
PCTV-1	V59.4 (%)	98.00 (97.00-99.00)	98.00 (96.50-99.00)	98.85 (98.40-99.56)	0.011	1	0.192	0.016
	D2 (Gy)	75.82 (75.57-76.32)	76.26 (74.37-78.37)	75.69 (74.62-76.60)	0.867	_	_	_
	D50 (Gy)	72.05 (71.83-72.32)	72.06 (71.49-72.89)	72.59 (70.09-73.73)	0.097	_	_	_
	D95 (Gy)	62.35 (61.888-63.05)	62.42 (60.52-63.45)	63.02 (62.41-64.26)	0.172	_	_	_
	D98 (Gy)	59.34 (58.09-60.33)	58.58 (56.84-60.44)	60.97 (60.28-62.33)	0.002	1	0.021	0.003
	CI	0.360 (0.330-0.405)	0.380 (0.340-0.445)	0.430 (0.385-0.480)	<0.001	0.228	<0.001	0.021
PCTV-2	V54 (%)	97.00 (95.50-98.00)	97.00 (96.00-98.00)	98.27 (96.42-98.98)	0.003	1	0.005	0.05
	D2 (Gy)	75.23 (74.87-75.62)	75.06 (73.45-77.32)	75.03 (74.23-76.17)	0.717	_	_	_
	D50 (Gy)	65.15 (63.92-66.81)	65.72 (64.37-67.17)	64.24 (62.18-65.63)	0.002	1	0.026	0.002
	D95 (Gy)	55.11 (54.73-55.42)	54.92 (54.63-55.50)	55.65 (55.03-56.18)	0.044	1	0.228	0.017
	D98 (Gy)	52.97 (51.74-54.01)	52.86 (51.83-54.06)	54.45 (52.77-55.05)	0.007	0.84	0.135	0.006
	CI	0.690 (0.655-0.735)	0.760 (0.730-0.780)	0.730 (0.710-0.770)	<0.001	<0.001	0.003	0.948

IMRT/I, intensity modulated radiation therapy; VMAT/V, volumetric modulated arc therapy; TOMO/T, tomotherapy; Gy, gray; IQR, inter-quartile range; DV, the absorbed dose in v% of the volume; CI, conformity index; HI, homogeneity index; Vx, the volume of organ receiving more or equal to x Gy.

**Table 9:** Comparison of organs at risk (priority level I and II) in T_3-4_ NPC patients.

		Median (IQR)				P-value		
OAR	Objective	IMRT	VMAT	TOMO	P	I *vs.* V	I *vs.* T	V *vs.* T
Brain stem	D_max_ (Gy)	54.26 (51.68-56.47)	55.50 (51.27-58.72)	52.50 (48.36-55.46)	0.005	0.495	0.192	0.004
Brain stem_PRV	D_0.03cc_ (Gy)	56.99 (54.26-63.71)	62.13 (54.95-64.87)	60.21 (55.83-64.81)	0.867	_	_	_
Optic chiasm	D_max_ (Gy)	44.79 (37.66-48.40)	41.65 (31.82-49.91)	45.82 (34.86-48.35)	0.717	_	_	_
Optic chiasma_PRV	D_0.03cc_ (Gy)	53.12 (45.23-55.99)	50.96 (47.05-57.14)	54.13 (46.59-56.64)	0.538	_	_	_
Optic nerves_L	D_max_ (Gy)	38.28 (27.50-45.20)	37.43 (27.82-45.55)	42.61 (37.29-44.40)	0.06	_	_	_
Optic nerves_L PRV	D_0.03cc_ (Gy)	46.24 (33.09-52.49)	45.42 (35.24-56.66)	51.90 (46.09-54.34)	0.005	1	0.041	0.006
Optic nerves_R	D_max_ (Gy)	40.45 (25.32-50.77)	39.24 (27.17-49.94)	43.89 (35.92-50.30)	0.156	_	_	_
Optic nerves_R PRV	D_0.03cc_ (Gy)	47.91 (35.70-57.67)	49.90 (39.98-56.14)	53.11 (45.32-54.95)	0.129	_	_	_
Spinal cord	D_max_ (Gy)	39.34 (38.42-40.50)	41.84 (39.15-43.69)	37.00 (32.38-40.78)	0.001	0.092	0.269	<0.001
Spinal cord_PRV	D_0.03cc_ (Gy)	45.38 (41.93-49.18)	46.66 (43.71-51.37)	44.76 (41.06-52.05)	0.467	_	_	_
Temporal lobe_L	D_max_ (Gy)	77.01 (76.41-78.28)	78.06 (75.01-80.22)	75.54 (73.77-76.74)	0.002	1	0.016	0.004
Temporal lobe_L PRV	D_0.03cc_ (Gy)	76.95 (76.26-77.96)	78.27 (75.73-80.28)	75.73 (74.91-77.05)	0.003	0.84	0.076	0.003
Temporal lobe_R	D_max_ (Gy)	77.46 (77.06-79.48)	77.75 (75.42-80.80)	75.40 (74.56-77.26)	0.001	1	0.004	0.004
Temporal lobe_R PRV	D_0.03cc_ (Gy)	77.55 (77.02-78.91)	79.02 (75.82-80.54)	75.48 (74.58-77.19)	0.013	1	0.033	0.033

IMRT/I, intensity modulated radiation therapy; VMAT/V, volumetric modulated arc therapy; TOMO/T, tomotherapy; OAR, organ at risk; IQR, inter-quartile range; Gy, gray; PRV, planning risk volume; L, left; R, right; Dmax, the maximum dose; D0.03cc, an approximate maximum dose for the organs at risk.

Subgroup analysis in patients with stage T_3-4_ disease showed that the median V69.96 for the IMRT, VMAT and TOMO plans was 90%, 91% and 93.61%, respectively. Although none of the plans achieved 95%, the TOMO plan was the highest, and a significant difference was observed only between the TOMO and IMRT plans (P = 0.033). In addition, the median V59.4 for IMRT, VMAT and TOMO was more than 98%, with the TOMO plan being the highest (P = 0.011).

In patients with stage T_3-4_ disease, the HI, CI, D2, D50 and D98 of PGTVnx among the three plans were not significantly different, except for the D95 of the PGTVnx which was the higher in the TOMO plan than in the IMRT plan. Additionally, the relative volumes of V59.4 and V54 in the TOMO plan were the highest. TOMO performed significantly the best regarding CI of PCTV-1. The D2, D50 and D95 of PCTV-1 were similar among the three plans.

Regarding D_max_ of brain stem, D_max_ of the spinal cord and the temporal lobe, the values with the TOMO plan were significantly lower than those with the VMAT plan. IMRT and VMAT were equally capable of sparing of most OARs, such as the brain stem, optic nerve, optic chiasma, spinal cord, temporal lobe, eye, pituitary glands, parotid gland, mandible and oral cavity. Compared to the VMAT plan, the TOMO plan appeared to be better for the sparing of the brain stem, spinal cord and temporal lobe.

As was shown in the **Table 10**, the low-dose volume of healthy tissue was significantly the lowest in the TOMO plan regarding V5. When comparing the median V10, V15, V20 and V25 values among the three plans, the TOMO plan values were significantly higher than the IMRT and VMAT values.

**Table 10:** Low-dose radiation volume of IMRT, VMAT and TOMO in T_3-4_ NPC patients.

		Median (IQR)				P-value		
OAR	Objective	IMRT (cm^3^)	VMAT (cm^3^)	TOMO (cm^3^)	P	I vs. V	I *vs.* T	V *vs.* T
Body	V5	7006 (5660-7460)	7094 (5634-7440)	6578 (5586-7217)	0.006	1	0.016	0.016
	V10	5899 (4937-6415)	5959 (4944-6436)	6037 (5087-6669)	<0.001	0.495	0.016	<0.001
	V15	5316 (4419-5778)	5337 (4405-5788)	5386 (4370-6134)	<0.001	1	0.001	<0.001
	V20	4764 (3904-5193)	4771 (3929-5204)	4950 (3849-5532)	<0.001	1	0.006	<0.001
	V25	4351 (3519-4734)	4254 (3557-4665)	4472 (3539-5048)	<0.001	0.062	0.192	<0.001
	V30	3920 (3141-4290)	3768 (3147-4098)	3856 (3102-4409)	0.004	0.004	1	0.062

IMRT/I, intensity modulated radiation therapy; VMAT/V, volumetric modulated arc therapy; TOMO/T, tomotherapy; OAR, organ at risk; IQR, inter-quartile range; Vx, the volume of organ receiving more or equal to x Gy.

## Discussion

With improvements in radiation techniques, local control of NPC has been greatly enhanced through the wide application of IMRT, VMAT and TOMO. Nevertheless, it remains unclear which kind of radiation technique is best for NPC. Therefore, we aimed to explore which technique benefits PTVs the most and resulted in the lowest absorbed dose in the OARs. More importantly, when we optimized all the plans according to the latest recommended guidelines (15) recommended, the PTV coverage was set as priority II, lower than the critical OARs classified as priority I.

Our research demonstrated that the IMRT plan showed the worst PTVs coverage and failed to meet the prescribed requirement. The dose coverage of the PGTVnx with the three radiation techniques in patients with advanced T stages NPC was unsatisfactory because the priority of the target dose coverage was lower than that of critical OARs (the brain stem, spinal cord, optic chiasma and optic nerves). The results from another small sample study were somewhat similar to ours, indicating that the target coverage volume of the TOMO plan is higher than that of the IMRT plan (97% *vs.* 94.3%, P < 0.05) (12).

However, the research conducted by Sun et al. (13) showed that both the IMRT and VMAT plans achieved 96.2% of the PTV covered by 7000 cGy of the prescription dose at the expense of the brain stem, optic chiasma, optic nerves and spinal cord. The maximum acceptable prescription dose of the brain stem was 54 Gy, but the pass rates of both the IMRT and VMAT plans were 28.8% (15/52) and 32.7% (17/52), respectively. In addition, the highest pass rates of the optic nerve, optic chiasma and spinal cord were only 65.4%, 53.8% and 80.8%, respectively.

The factors contributing to these contradictory results may be the different priorities of the target dose. Sun’s study was designed to protect critical OARs as much as possible on the basis of meeting the prescribed dose of PTVs, thus causing a lower pass rate of critical OARs and a higher coverage volume of prescribed dose for PTVs. Radiotherapy is a double-edged sword that can both kill tumor cells and damage normal tissues. Only by properly balancing the dose of the tumor target can radiotherapy achieve the maximum effect. Hence, the newest guidelines published in 2019 (15) recommends that the safety of the treatment for patients should be taken into consideration first, which means that the dose limitation of OARs classified as priority I (such as the brain stem, spinal cord, optic chiasma and optic nerves) should be considered first and the dose coverage of PTVs should be considered second.

Our research showed that the VMAT plan was comparable to the IMRT plan in terms of the CI and HI of the PGTVnx. The results were inconsistent with those observed in another study comparing the VMAT plan with the IMRT plan, which showed similar conformity and dose homogeneity for high-dose targets (18). Noticeably, the small sample size of patients in this similar study may have created considerable selection bias.

Our results showed that the D2 of the PTVs were the same among the three plans. The results are in contrast with those observed in the latest study conducted by He et al. (9), which showed that compared to the VMAT plan, the IMRT plan significantly increases the D2 of the PTVs (PGTVnx: 78.07 Gy *vs.* 76.86 Gy, P < 0.01; PCTV-1: 77.54 Gy *vs.* 76.68 Gy, P < 0.01; PCTV-2: 76.46 Gy *vs.* 75.49 Gy, P < 0.01). Another study indicated that the IMRT plan significantly increased the D2 of the PTVs which was only observed in patients with early-stage tumors (7564 cGy ± 92 cGy *vs.* 7494 cGy ± 109 cGy, P = 0.016) (19). The possible for this may be that IMRT increases the dose of the PTVs as much as possible by increasing the dose of hot spots after achieving the dose limitation of critical OARs such as the brain stem, optic chiasma and spinal cord. However, a study published in 2013 demonstrated the opposite result, showing that the D2 of PTVs was higher with the VMAT plan than with the IMRT plan (13).

The greatest difficulty with radiotherapy for NPC is that the primary tumor is adjacent to many critical OARs, which limits the radiation dose delivered. A study conducted by He et al. (9) revealed that late toxicities of radiotherapy were related to the dose absorbed by the corresponding OARs. Thus, a desirable plan balances the delivery of a high dose to the PTVs and a low dose to the OARs as much as possible.

Another highlight of our research is that the dose acceptance criteria of OARs in radiation therapy planning for NPC obeyed the newest international guidelines (15). We noticed that the limitation criteria of OARs in nearly all published studies focusing on the comparison of different radiation technologies in patients with NPC were based on RTOG0615, published in 2011. The dose limitation summarized in RTOG0615 was derived from two-dimensional radiotherapy approaches ten years ago and is not fully applicable to the currently used intensity-modulated radiation therapies. The newest OARs limitation guidelines indicated that the MACs for the brain stem, optic nerves and optic chiasma are 60 Gy; those in the RTOG0615 were 54 Gy, 50 Gy and 50 Gy. The increase in the safety limitation dose of these critical OARs ranges from 6 Gy to 10 Gy, which plays an important role in improving the local control of NPC with advanced radiation techniques.

Our research indicates that each of the three advanced radiation techniques has advantages and disadvantages regarding the protection of OARs.

Two previous studies demonstrated that the TOMO plan was significantly superior to the IMRT plan regarding the brain stem, spinal cord and optic nerves (P <0.05) (11, 12). Another study has shown that the VMAT plan leads to a higher absorbed dose than the IMRT plan in the brain stem and spinal cord, especially in patients with early-stage disease (19). Nonetheless, comparable results regarding protection of the brain stem and spinal cord between the VMAT and IMRT plans were reported by Johnston et al. (20) and Fung et al. (10).

In patients with early T-stage disease, the advantages of the TOMO plan were not obvious, and the TOMO plan was even inferior to the VMAT or IMRT plan in sparing the optic chiasma, optic nerves and pituitary gland. Moreover, the low-dose radiation volume of the TOMO plan was the highest among three plans, especially for V5, V10, V15 and V20. The TOMO plan also achieved a lower dose than the VMAT plan regarding the brain stem, spinal cord and temporal lobes. The results of another study that enrolled patients with early T-stage NPC were strikingly similar to ours (21). In addition, the coast of TOMO for patients is higher than that of VMAT in clinical practice.

In patients with advanced T-stage disease, the dose coverage of V69.96 in the TOMO plan was the highest, and reached 93.61%. We also found that no significant difference was observer among the IMRT, VMAT and TOMO plans with regard to sparing the optic chiasma and optic nerves. However, another study showed that VMAT was inferior to IMRT for protecting critical structures, which was completely contrary to our conclusion (13). One possible reason may be that the dose constraint of OARs classified as priority I (such as the optic chiasma and optic nerves) was satisfied first in our study. Additionally, our study demonstrated that TOMO achieved the best sparing of the brain stem, spinal cord and temporal lobes when compared to that achieved with IMRT and VMAT in advanced-T-stage patients. Regardless, published studies focusing on patients with advanced T-stage NPC are rare. Hence, our results need to be validated with large randomized controlled trials.

## Conclusion

In the early T stage, the IMRT, VMAT, and TOMO plans achieved ideal dose coverage of the targets. The TOMO plan, with a higher volume of low-dose radiation, had no significant advantages in most of the OARs protection. Thus, there were no obvious advantages to choosing the TOMO plan for patients with early T stage NPC. In addition, the VMAT plan provides similar dose coverage and OARs protection compared to those achieved with the IMRT plan. The heterogeneity index (HI) of the PGTVnx with the VMAT plan was better than that with the IMRT plan.

For patients with advanced T stage NPC, neither the IMRT plan nor the VMAT or TOMO plan reached a 100% prescription dose covering more than 95% of the PGTVnx, however, the TOMO plan achieved the largest dose coverage of the PGTVnx. Additionally, the TOMO plan could better protect the brain stem, spinal cord and temporal lobe. Therefore, the TOMO plan may be recommended for patients with advanced T stage NPC.

## Limitations

The limitation of our research should be noted. This was designed as a retrospective analysis. Hence, further investigation is needed to determine whether our results can be translated into clinical advantages.

## Data Availability Statement

The original contributions presented in the study are included in the article/**Supplementary Material**. Further inquiries can be directed to the corresponding authors.

## Ethics Statement

The studies involving human participants were reviewed and approved by Ethics Committee of Yunnan Cancer Hospital. The patients/participants provided their written informed consent to participate in this study.

## Author Contributions

All the authors listed contributed to this research and the specific tasks undertaken by the individual are as follows. WX, WL, and WJ: study design. QW: manuscript drafting. JQ: communication and coordination of the implementation process. RC: collection of statistics. TX: data analysis. JW, GX, and LZ: manuscript revision. JY: planning. SZ: plans evaluation. QW, JQ, RC and TX contributed equally to this work. All authors contributed to the article and approved the submitted version.

## Funding

1. “Ten Thousand Plan” Youth Talent Project in Yunnan Province (no grant number is applicable). 2. Scientific Research Fund Project of the Yunnan Provincial Department of Education. (Grant number: 2018JS222). 3. Cancer Radiation Therapy Technology Innovation Team Construction Project Funding of Kunming Medical University (Grant number: CXTD201806). 4. Health Science and Technology Program of Yunnan Province (Grant number: 2018NS0066).

## Conflict of Interest

The authors declare that the research was conducted in the absence of any commercial or financial relationships that could be construed as a potential conflict of interest.

The reviewer [YT] declared a shared affiliation with one of the authors [WJ] to the handling editor at the time of review.

## Publisher’s Note

All claims expressed in this article are solely those of the authors and do not necessarily represent those of their affiliated organizations, or those of the publisher, the editors and the reviewers. Any product that may be evaluated in this article, or claim that may be made by its manufacturer, is not guaranteed or endorsed by the publisher.
